# Quantification of Ki67 Change as a Valid Prognostic Indicator of Luminal B Type Breast Cancer After Neoadjuvant Therapy

**DOI:** 10.3389/pore.2021.1609972

**Published:** 2021-12-20

**Authors:** Shirong Tan, Xin Fu, Shouping Xu, Pengfei Qiu, Zhidong Lv, Yingying Xu, Qiang Zhang

**Affiliations:** ^1^ Department of Breast Surgery, The First Affiliated Hospital of China Medical University, Shenyang, China; ^2^ Department of Breast Surgery, Cancer Hospital of China Medical University, Liaoning Cancer Hospital & Institute, Shenyang, China; ^3^ Department of Breast Surgery, Harbin Medical University Cancer Hospital, Harbin, China; ^4^ Breast Cancer Center, Shandong Cancer Hospital and Institute, Shandong First Medical University and Shandong Academy of Medical Sciences, Jinan, China; ^5^ Breast Center, The Affiliated Hospital of Qingdao University, Qingdao, China

**Keywords:** breast cancer, prognosis, neoadjuvant chemotherapy, Ki67, tumor response

## Abstract

**Introduction:** Ki67 value and its variation before and after neoadjuvant chemotherapy are commonly tested in relation to breast cancer patient prognosis. This study aims to quantify the extent of changes in Ki67 proliferation pre- and post-neoadjuvant chemotherapy, confirm an optimal cut-off point, and evaluate its potential value for predicting survival outcomes in patients with different molecular subtypes of breast cancer.

**Methods:** This retrospective real-world study recruited 828 patients at the Department of Breast Surgery of the First Affiliated Hospital of China Medical University and the Cancer Hospital of China Medical University from Jan 2014 to Nov 2020. Patient demographic features and disease pathology characteristics were recorded, and biomarkers were verified through immunohistochemistry. Various statistical methods were used to validate the relationships between different characteristics and survival outcomes irrespective of disease-free and overall survival.

**Results:** Among 828 patients, statistically significant effects between pathological complete response and survival outcome were found in both HER2-enriched and triple-negative breast cancer (*p* < 0.05) but not in Luminal breast cancer (*p* > 0.05). Evident decrease of Ki67 was confirmed after neoadjuvant chemotherapy. To quantify the extent of Ki67 changes between pre- and post-NAC timepoints, we adopted a computational equation termed *ΔKi67%* for research. We found the optimal cut-off value to be “*ΔKi67%* = −63%” *via* the operating characteristic curve, defining *ΔKi67%* ≤ −63% as positive status and *ΔKi67%* > −63% as negative status. Patients with positive *ΔKi67%* status were 37.1% of the entire cohort. Additionally, 4.7, 39.9, 34.5 and 39.6% of patients with Luminal A, Luminal B, HER2-enriched and triple negative breast cancer were also validated with positive *ΔKi67%* status. The statistically significant differences between *ΔKi67%* status and prognostic outcomes were confirmed by univariate and multivariate analysis in Luminal B (univariate and multivariate analysis: *p* < 0.05) and triple negative breast cancer (univariate and multivariate analysis: *p* < 0.05). We proved *ΔKi67%* as a statistically significant independent prognostic factor irrespective of disease-free or overall survival among patients with Luminal B and triple-negative breast cancer.

**Conclusions:** *ΔKi67%* can aid in predicting patient prognostic outcome, provide a measurement of NAC efficacy, and assist in further clinical decisions, especially for patients with Luminal B breast cancer.

## Introduction

Breast cancer is the highest cause of cancer-related morbidity among women worldwide [1]. Established biomarkers, including hormone receptors (HR), estrogen receptor (ER), progesterone receptor (PR), human epidermal growth receptor-2(HER-2), and Ki67 labeling index classify breast cancer into four subtypes: HER2-enriched, triple-negative (TN), and Luminal A and B types [2,3]. Neoadjuvant chemotherapy (NAC) is a standard therapeutic strategy for inoperable breast cancer and for some operable patients who seek decreased primary tumor burden and breast conservation [4]. Patient response to NAC also provides guidance for the long-term systemic therapeutic strategy for each individual patient [5]. A achievement of pathological complete response (pCR), disease-free survival (DFS), and overall survival (OS) [6] were used to estimate treatment efficacy. Only 15–20% of patients who receive NAC reach pCR [7–9]. Although pCR plays an important role in prognostic prediction and assists in treatment decisions for TN and HER2-enriched breast cancer, it is less effective in Luminal breast cancer subtypes [10–12]. Luminal breast cancer still lacks indicators to classify patients who will benefit from NAC.

Ki67, a nuclear indicator of cellular proliferation, has been extensively studied and scrutinized for several years. Although some studies criticize Ki67 for its lack of reproducibility [13,14], many demonstrate that proliferation index relates to patient outcomes [2,4,15–17]. These study showKi67 expression as useful indicator for breast cancer and a useful prognostic factor for patients with Luminal B and node-positive breast cancer, assisting in clinical decision regarding neoadjuvant endocrine therapy [18,19]. Some studies indicates that Ki67 levels pre-NAC can be an independent prognostic predictor for OS and DFS [12,16]. Endocrine therapy can decrease cell proliferation, presenting as changed Ki67 level pre- and post-NAC [20,21]. In the POETIC clinical phase-3 trial, this change in Ki67 levels was able to guide endocrine therapy decisions for women with ER-positive breast cancer [19]. One commonality across studies was that decreased levels of Ki67 post-NAC compared to pre-NAC holds significant prognostic predictive value [12,18,22–25]. However, some researchers contend that post-NAC Ki67 may hold limited prognostic value [26].

Previous researches often define the extent of Ki67 change between pre- and post-NAC simply by subtracting the two values. This definition is simple but insufficient, as illustrated in two scenarios. The first is if pre- and post-NAC Ki67 proliferation are both relatively low, then the extent of the change may not reach the set threshold. In the second, the change may be comparatively large but not large enough to reach the cut-off value. Furthermore, high variation of pre- and post-NAC Ki67 have been classified by several groups, with studies proposing different thresholds of variation based on the attempts.

In this retrospective study, we evaluate the usefulness of Ki67 change before and after NAC for predicting survival outcome across breast cancer molecular subtypes. We further quantify the change in Ki67 by percentage before and after NAC and calculate an optimal threshold to assess its predictive function for long-term survival and its ability to aid in deciding further adjuvant therapy modification across breast cancer subtypes.

## Methods

### Patient Selection Criterion

This retrospective study included patients with primary breast cancer who were treated with NAC from Jan 2014 to Nov 2020 at the Department of Breast Surgery of the First Affiliated Hospital of China Medical University and the Cancer Hospital of China Medical University.

### Patient Inclusion Criteria

All patients received a minimum of one cycle of NAC ahead of surgery. Patients with cancer *in situ* were excluded, as were patients with invasive breast cancer before NAC could be incorporated in cohort. Patients who received any kind of treatment prior to NAC or who presented with progressive or metastatic breast cancer were excluded. Patients with previous breast cancer, male patients, and those with synchronous invasion, bilateral, or inflammatory breast cancer were also excluded. 68 cases with incomplete or deficient IHC analysis were also excluded. In total, 828 patients met the above restriction standards and were included.

### Classifications of Patients

This retrospective study received permission from the institutional review board (IRB) of the First Affiliated Hospital and was in accordance with the Helsinki Declaration. All patients involved in the research gave informed consent in written agreements of specimens used for scientific research. The informed consent of retrospective research involvement could be waived based on the retrospective nature of the study. Pre-NAC core needle biopsy pathology and post-surgery regular pathology was extracted and saved in a database. Patient characteristics were collected including gender, age at diagnosis, body mass index (BMI), maximum tumor diameter, tumor grade and stage, axillary lymph node status, histologic type, NAC schedule and cycle number, histology grade, and clinical response to NAC. The local extent of breast cancer was measured *via* breast ultrasound, mammography, breast MRI, chest CT, bone scan and/or hepatobiliary and splenic ultrasound to verify distant metastasis. The final size of local breast cancer in our database was adopted following priorities: breast MRI > breast ultrasound > mammography. Every patient with suspicious lymph-node metastasis suggested by imaging examinations underwent ultrasound-guided core biopsy of ultrasound-graphically abnormal nodes for axillary node metastasis confirmation before starting NAC. The final histological assessments were all analyzed using hematoxylin and eosin staining and immunohistochemical (IHC). The histological type of specimens from incorporated patients was distinguished between two subgroups: general invasive breast carcinoma of no special type (IBC-NST) and Others. The latter group contained special subtypes such as lobular, mucinous and tubular carcinomas with ≥90% of the tumor as a pure special tumor type and mixed IBC-NST with special subtypes. Lymph nodes with micro-metastasis were considered positive, including the maximum diameter over 0.2 mm or the number of tumor cells over 200. Isolated tumor cells were considered negative.

### Assessment of Clinical Effectiveness of Chemotherapy

Pathological complete response (pCR) was defined as no residual invasive tumor upon hematoxylin and eosin evaluation of the complete resected breast specimen and all sampled lymph nodes (noninvasive breast residuals) (ypT0/is, ypN0).

### Immunohistochemistry for Biomarker Detection

Histopathology is regarded as the gold standard for diagnosis. All breast tumor specimens were acquired from core needle biopsy or surgical resections, and every specimen was affixed into formalin-fixed, paraffin-embedded tissue sections for preservation. IHC staining was performed using Dako Autostainer Plus and EnVision Dual Link detection reagent (DAKO; Carpinteria, CA) with DAB (Dako). Biomarker status, including ER, PR and HER2, were defined by IHC in strict accordance with European Quality Assurance guidelines. ER and PR staining were assessed based on the American Society of Clinical Oncology/College of American Pathologists Guidelines [27,28]. Antibodies used in IHC include as anti-ER (Clone SP1, Dako), anti-PR (Clone PgR636, Dako), and Ki67 (Clone Mib-1, Dako). Hematoxylin II (Dako As Link 48) was used to counterstain specimens automatically. All tests incorporated external positive and negative controls. ER and PR stains were considered positive if immunostaining was seen in more than 1% of immunoreactive cells. HER2 status was ascertained *via* IHC using the Hercep Test ™kit (code K5204, Dako). HER2 expression was scored as 0, 1+, 2+, and 3 + according to ASCO guidelines. A score of 3+ was regarded as HER2+, with 0/1+ defined as HER2-. For cases scoring HER2 2+, a fluorescent *in situ* hybridization (FISH) test could be conducted. The measurement of Ki67 index was based on the spot with the highest intensity in a high-power field (400x) and 500–2000 cells were counted [29].

Following the St. Gallen guidelines 2013 [2], high expression of PR was set as ≥20% and low expression of PR was defined as <20%. In accordance with the International Ki67 in Breast Cancer Working Group [30], Ki67 index was classified into two groups: low (<30%), and high (≥30%). To quantify the extent of Ki67 changes between pre- and post-NAC timepoints, we used the following equation: [define post-NAC Ki67 as A, define pre-NAC Ki67 as B, computational formula: *ΔKi67%*=(A−B)/B × 100%, maintaining sign]. If a patient achieved pCR after NAC, the post-NAC Ki67 index was defined as 0% and *ΔKi67%* was mathematically −100%. Representative IHC staining images of Ki67 subgroups are shown in Supplementary Figure S2.

### Breast Cancer Subtypes Definitions

We classified breast cancers into four subtypes based on HR status, HER2 status and Ki67 index according to the St. Gallen guidelines as follow [2,31]: Luminal A: (ER and PR positive, HER2 negative, “low” Ki-67, and a “low” recurrence risk based on multi-gene-expression assay results if available), Luminal B [“Luminal B-like (HER2 negative)”: ER positive, HER2 negative, and at least one of the following: “high” Ki-67, “negative or low” PR, or “high” recurrence risk based on multi-gene-expression assay if available. “Luminal B-like (HER2 positive)”: ER positive, HER2 over-expressed or amplified with any Ki-67, and any PR]. HER2-enriched (HER2 over-expressed or amplified, ER and PR absent) and TN (Negative ER, PR and HER2). The cut-point between “high” and “low” values for Ki67 varies and lacks conclusion. 14% cut-off value of Ki67 for subtype classification was adopted in St. Gallen guidelines 2013.

### Statistical Data Analysis

Multiple demographic features were analyzed using the Chi-square test. The survival-related indicators studied were DFS and OS. DFS was calculated from the date of initiation of the first regimen to the date of first event (locoregional relapse, distant relapse, or death) and OS was calculated from the date of surgery to the date of death or last follow-up. The Kaplan–Meier method was used to define the difference ratio, and survival curves were compared using the log-rank test [32]. Significance was assigned as *p* value < 0.05. Receiver operating characteristic curve (ROC) and area under the curve (AUC) were performed to calculate the optimal cut-off value determined by the Youden index with maximum sensitivity and specificity. Cox proportional hazards regression models were used to estimate relapse and survival risk between subgroups. The multivariate Cox proportional hazards model was implemented for Hazard Ratio (HR) and 95% confidence intervals (CI) to identify independent prognostic factors. Net reclassification improvement (NRI) was used to verify classification accuracy. All statistical data analysis was performed using SPSS 26.0 (SPSS Inc., Chicago, IL, United States) and R programming language (version 3.5.3; https://www.r-project.org/).

## Results

### Basic Demographic Features and Baseline Characteristics

**FIGURE 1:**
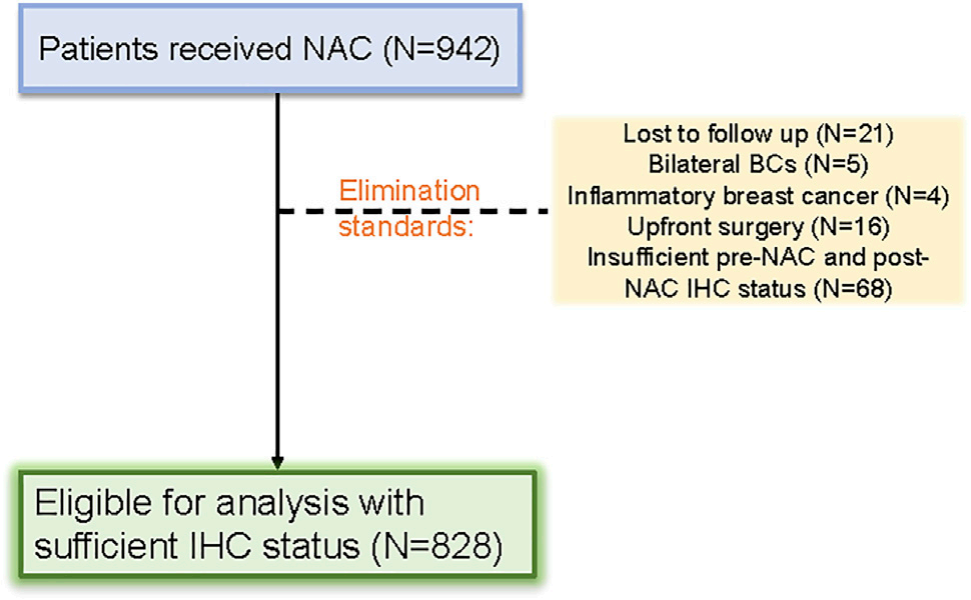
Patient selection flow diagram for the study.

Patients with primary breast cancer who were treated with NAC were selected based on strict standards. 942 patients were initially included in the cohort, but 114 patients were eliminated for various reasons. A total of 828 female patients with primary breast cancer who received NAC were ultimately included in this retrospective study (Figure 1).

Basic demographic and pathologic features are shown in Table 1. The median age of entire cohort was 51 ± 9.65 years old (range: 23–76 years), of which 10.0% of patients were under 40 years old at diagnosis. Body mass index was used to distinguish subjects: overweight patients with index greater than or equal to 24.9 accounted for 48.2%, underweight patients with index under 18.9 accounted for 8.2 and 43.6% of patients had a healthy BMI between 18.9–24.9. Breast cancer pathological subtype was confirmed as invasive carcinoma of NST for 84.4% of patients. All other histological types represented 15.6% of the full cohort. A median of 4 NAC cycles were received (range: 1–9), with NAC classified into three groups: texane-based (10.7%), anthracycline-based (18.1%) and texane + anthracycline (71.1%). The average maximum tumor diameters before and after NAC were 3.41 ± 1.651 and 2.26 ± 1.648 cm, respectively. 81.9% patients presented with node-positive status at diagnosis. Finally, 138 subjects (16.7%) who received NAC achieved pCR, a commonly used measurement of NAC efficacy.

**TABLE 1:** Demographic and clinicopathological features of whole cohort (*n* = 828).

Parameter	Number (%)
Age at diagnosis (year)	
<40	83 (10.0)
≥40	745 (90.0)
BMI (kg/m^2^)	
<18.9 (underweight)	68 (8.2)
18.9–24.9	361 (43.6)
>24.9 (overweight)	399 (48.2)
Histological type at diagnosis	
IBC-NST	699 (84.4)
Others	129 (15.6)
Clinical nodal status at diagnosis	
Positive	676 (81.6)
Negative	152 (18.4)
Chemotherapy cycles	
≤2	199 (24)
3–5	424 (51.2)
>5	205 (24.8)
Chemotherapy regimen	
Taxane -based	89 (10.7)
Anthracycline-based	150 (18.1)
Taxane + anthracycline	589 (71.1)
Anti-HER2 therapy in patients with HER2-positive (*n* = 261)	
Yes	49 (18.8)
No	212 (81.2)
Clinical tumor stage at diagnosis	
T1	80 (9.7)
T2	556 (67.1)
T3/T4	192 (23.2)
Post-NAC tumor size	
<2 cm	434 (52.4)
2–5 cm	355 (42.9)
>5 cm	39 (4.7)
Response to NAC	
PR/CR	494 (59.7)
SD/PD	334 (40.3)
Achieved pCR	
Yes	138 (16.7)
No	690 (83.3)
ER status^a^	
Positive	526 (63.5)
Negative	302 (36.5)
PR positivity score^b^	
<20%	520 (62.8)
≥20%	308 (37.2)
HER2	
Positive	261 (31.5)
Negative	447 (54.0)
Unknown	120 (14.5)
Pre-NAC Ki67	
<30%	253 (30.6)
≥30%	575 (69.4)
Post-NAC Ki67	
<30%	491 (59.3)
≥30%	337 (40.7)
Molecular subtypes^c^	
Luminal A	43 (5.2)
Luminal B	489 (59.1)
HER2-enriched	148 (17.9)
TNBC	148 (17.9)

a Positivity score<1% including negative status.

b Positivity score<20% including negative status.

c Luminal A: (ER and PR positive, HER2 negative, “low” Ki-67, and a “low” recurrence risk based on multi-gene-expression assay results if available), Luminal B (“Luminal B-like (HER2 negative)”: ER positive, HER2 negative, and at least one of the following: “high” Ki-67, “negative or low” PR, or “high” recurrence risk based on multi-gene-expression assay if available. “Luminal B-like (HER2 positive)”: ER positive, HER2 over-expressed or amplified with any Ki-67, and any PR). HER2-enriched (HER2 over-expressed or amplified, HR absent) and TN (Negative HR and HER2).

BMI, body mass index; NAC, neoadjuvant chemotherapy; IBC-NST, invasive breast carcinoma of no special Type; pCR, pathological complete response; ER, estrogen receptor; PR, progesterone receptor; HER2, human epidermal growth factor receptor 2; TNBC, triple negative breast cancer.

We next analyzed IHC biomarkers. Patients with ER positivity made up 59.5% of the cohort. Patients with PR < 20%, or negative status, represented 62.8% of the cohort. Based on strict IHC staining, subjects positive for HER2 accounted for 31.5% of cases 87.6% of all cases had Ki67 expression ≥30% before NAC, while only 58.9% of the cohort had ≥30% Ki67 after NAC. 18.8% of HER2-positive patients received anti-HER2 therapy.

Based on biomarker status, patients were categorized into four subtypes: 43 subjects (5.2%) were categorized as Luminal A subtype breast cancer, 489 subjects (59.1%) had a Luminal B breast cancer, 148 subjects (17.9%) had HER2-enriched breast cancer, and 148 cases (17.9%) had TN breast cancer. IHC status and subtypes distribution are shown in Table 1.

### Correlation Between Patient Features and Pathological Response to NAC or Survival

We chose pCR as our evaluation criterion of pathological response to NAC. The median follow-up time was 62.00 ± 21.43 months. A associations between patient features and pathological response to NAC or survival were assessed *via* the Chi-square test (χ2), with results shown in Table 2. Age, body mass index, maximum tumor diameter before NAC, and HER2 status all had *p* values > 0.05, indicating no significant influence on prognosis. However, different carcinoma pathology subtypes, maximum diameter after NAC, and nodal status at diagnosis were all significantly associated with pCR, with all *p* values < 0.05. The IHC biomarkers ER, PR and Ki67 status before and after NAC, all associated with pathological response to NAC. The log-rank test was used for in analysis of different parameters with DFS and OS fully considering the follow-up time (Table 3). For DFS and OS, BMI and Ki67 after NAC both presented *p* value < 0.05.

**TABLE 2:** The univariate relationship between above features with pCR (*n* = 828)

Parameter	Pathological response to NAC		
	pCR	Non-pCR	*p* Value
Age at prognosis (years)			0.196
<40	18	65	
≥40	120	625	
BMI (kg/m^2^)			0.539
<18.9	11	57	
18.9–24.9	66	295	
>24.9	61	338	
Histological type			<0.001
IBC-NST	95	604	
Others	43	86	
Chemotherapy cycles			0.432
≤2	30	169	
3–5	68	356	
>5	40	165	
Chemotherapy regimen			0.229
Taxane-based	13	76	
Anthracycline-based	32	118	
Taxane + anthracycline	93	496	
Anti-HER2 therapy in patients with HER2-positive (*n* = 261)			0.293
Yes	12	37	
No	38	174	
Clinical tumor stage at diagnosis			0.267
T1	14	66	
T2	85	471	
T3/T4	39	153	
Post-NAC tumor size			<0.001
<2 cm	104	330	
2–5 cm	30	325	
>5 cm	4	35	
Clinical nodal status			<0.001
Positive	99	577	
Negative	39	113	
ER status^a^			0.014
Positive	75	451	
Negative	63	239	
PR positivity score^b^			0.028
<20%	105	495	
≥20%	33	248	
HER2			0.259
Positive	50	211	
Negative	73	374	
Pre-NAC Ki67			0.147
<30%	35	218	
≥30%	103	472	
Post-NAC Ki67			<0.001
<30%	119	372	
≥30%	19	318	
Molecular subtypes^c^			0.025
Luminal A	3	40	
Luminal B	72	417	
HER2-enriched	29	119	
TNBC	34	114	

a Positivity score<1% including negative status.

b Positivity score<20% including negative status.

c Luminal A: (ER and PR positive, HER2 negative, “low” Ki-67, and a “low” recurrence risk based on multi-gene-expression assay results if available), Luminal B (“Luminal B-like (HER2 negative)”: ER positive, HER2 negative, and at least one of the following: “high” Ki-67, “negative or low” PR, or “high” recurrence risk based on multi-gene-expression assay if available. “Luminal B-like (HER2 positive)”: ER positive, HER2 over-expressed or amplified with any Ki-67, and any PR). HER2-enriched (HER2 over-expressed or amplified, HR absent) and TN (Negative HR and HER2).

BMI, body mass index; NAC, neoadjuvant chemotherapy; IBC-NST, invasive breast carcinoma of no special Type; pCR, pathological complete response; ER, estrogen receptor; PR, progesterone receptor; HER2, human epidermal growth factor receptor 2; TNBC, triple negative breast cancer.

**TABLE 3:** The univariate relationship between above features with DFS and OS (*n* = 828).

Parameter	DFS (*n* = 98)			OS (*n* = 59)		
	Events-free	Events	*p* Value	Events-free	Events	*p* Value
Age at prognosis (years)			0.299			0.181
<40	76	7		80	3	
≥40	654	91		689	56	
BMI (kg/m^2^)			**0.045**			**0.026**
<18.9	61	7		62	6	
18.9–24.9	323	38		344	17	
>24.9	346	53		363	36	
Histological type			0.849			0.382
IBC-NST	609	90		644	55	
Others	121	8		125	4	
Chemotherapy cycles			0.721			0.567
≤2	174	25		184	15	
3–5	377	47		397	27	
>5	179	26		188	17	
Chemotherapy regimen			0.204			0.364
Taxane-based	77	12		84	5	
Anthracycline-based	138	12		142	8	
Taxane + anthracycline	514	74		542	46	
Anti-HER2 therapy in patients with HER2-positive (*n* = 261)			0.771			0.712
Yes	44	5		47	2	
No	181	31		195	17	
Clinical tumor stage at diagnosis			0.889			0.499
T1	70	10		72	8	
T2	489	67		519	37	
T3/T4	171	21		178	14	
Post-NAC tumor size			0.871			0.778
<2 cm	384	50		404	30	
2–5 cm	311	44		328	27	
>5 cm	35	4		37	2	
Clinical nodal status			0.256			0.669
Positive	597	78		627	48	
Negative	132	20		141	11	
ER status^a^			0.377			0.293
Positive	471	55		494	32	
Negative	259	43		275	27	
PR positivity score^b^			0.246			0.059
<20%	449	71		474	46	
≥20%	281	27		295	13	
HER2			0.275			0.862
Positive	225	36		242	19	
Negative	402	45		417	30	
Unknown	103	17		110	10	
Pre-NAC Ki67			0.438			0.607
<30%	227	26		237	16	
≥30%	503	72		532	43	
Post-NAC Ki67			**0.008**			**0.004**
<30%	446	45		467	24	
≥30%	284	53		302	35	
Molecular subtypes^c^			0.335			0.571
Luminal A	42	1		42	1	
Luminal B	432	57		456	33	
HER2-enriched	128	20		137	11	
TNBC	128	20		134	14	

a Positivity score<1% including negative status.

b Positivity score<20% including negative status.

c Luminal A: (ER and PR positive, HER2 negative, “low” Ki-67, and a “low” recurrence risk based on multi-gene-expression assay results if available), Luminal B (“Luminal B-like (HER2 negative)”: ER positive, HER2 negative, and at least one of the following: “high” Ki-67, “negative or low” PR, or “high” recurrence risk based on multi-gene-expression assay if available. “Luminal B-like (HER2 positive)”: ER positive, HER2 over-expressed or amplified with any Ki-67, and any PR). HER2-enriched (HER2 over-expressed or amplified, HR absent) and TN (Negative HR and HER2).

BMI, body mass index; NAC, neoadjuvant chemotherapy; IBC-NST, invasive breast carcinoma of no special Type; pCR, pathological complete response; ER, estrogen receptor; PR, progesterone receptor; HER2, human epidermal growth factor receptor 2; TNBC, triple negative breast cancer.

**FIGURE 2:**
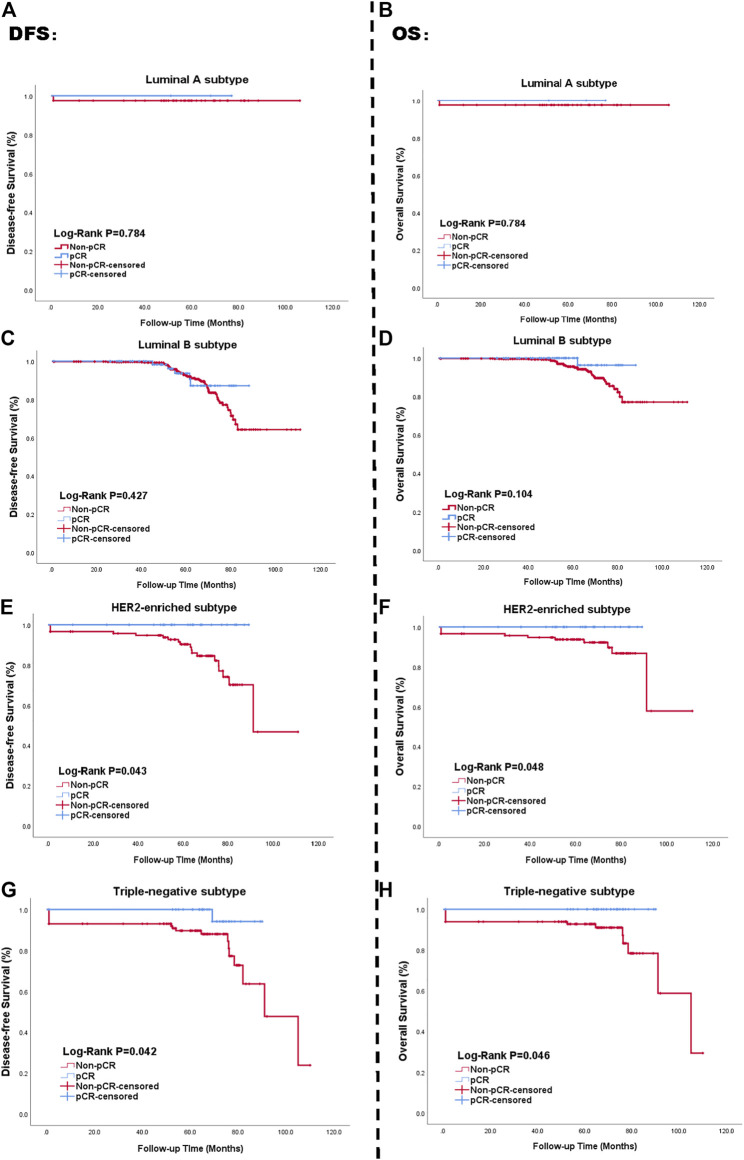
Kaplan-Meier curve of survival in patients with pCR status; Luminal A subtype **(A, B)**, Luminal B subtype **(C, D)**, HER2-enriched subtype **(E, F)**, TN breast cancer subtype **(G, H)**. Blue lines: achieving pCR; Red lines: not achieving pCR. The left side of figure represented the relationship between pCR status and DFS. The right side presented the relationship between pCR status and OS. Abbreviations: pCR, pathological complete response; HER2, human epidermal growth factor receptor 2; TN breast cancer, triple negative breast cancer; DFS, disease-free survival; OS, overall survival.

Histological subtype further affected both pCR rates and survival status. Each subtype resulted in different pathological responses, as validated by the Chi-square test (*p* < 0.05), but there was no obvious change on patient prognosis (*p* = 0.244 > 0.05). Rates of pCR across subtypes are shown in Figure 2. The left side of the picture presents the relation between pCR and DFS. The right side of the picture presents the univariate analysis of the relationship between OS and pCR based on the Kaplan-Meier method. We calculated survival rates for patients with each subtype using the Kaplan–Meier method. pCR status had no impact on DFS outcome for patients with Luminal A (*p* = 0.784 > 0.05, Figure 2A) or Luminal B subtypes (*p* = 0.427 > 0.05, Figure 2B). However, both HER2-enriched and TN breast cancer subtype patients showed a significant association between pathological response to NAC and survival outcome (*p* = 0.043 < 0.05, and *p* = 0.042 < 0.05, respectively, Figures 2C,D). The corresponding Kaplan-Meier curves of pCR with OS outcome for four subtypes are presented in Figures 2E–H. The multivariate Cox analysis for the full cohort is available in Supplementary Table S1. The visual result of univariate analysis displayed *via* Kaplan-Meier curves is in Supplementary Figure S1.

### Assessment of the Prognostic Efficacy of Ki67 Expression Status Before and After NAC

**FIGURE 3:**
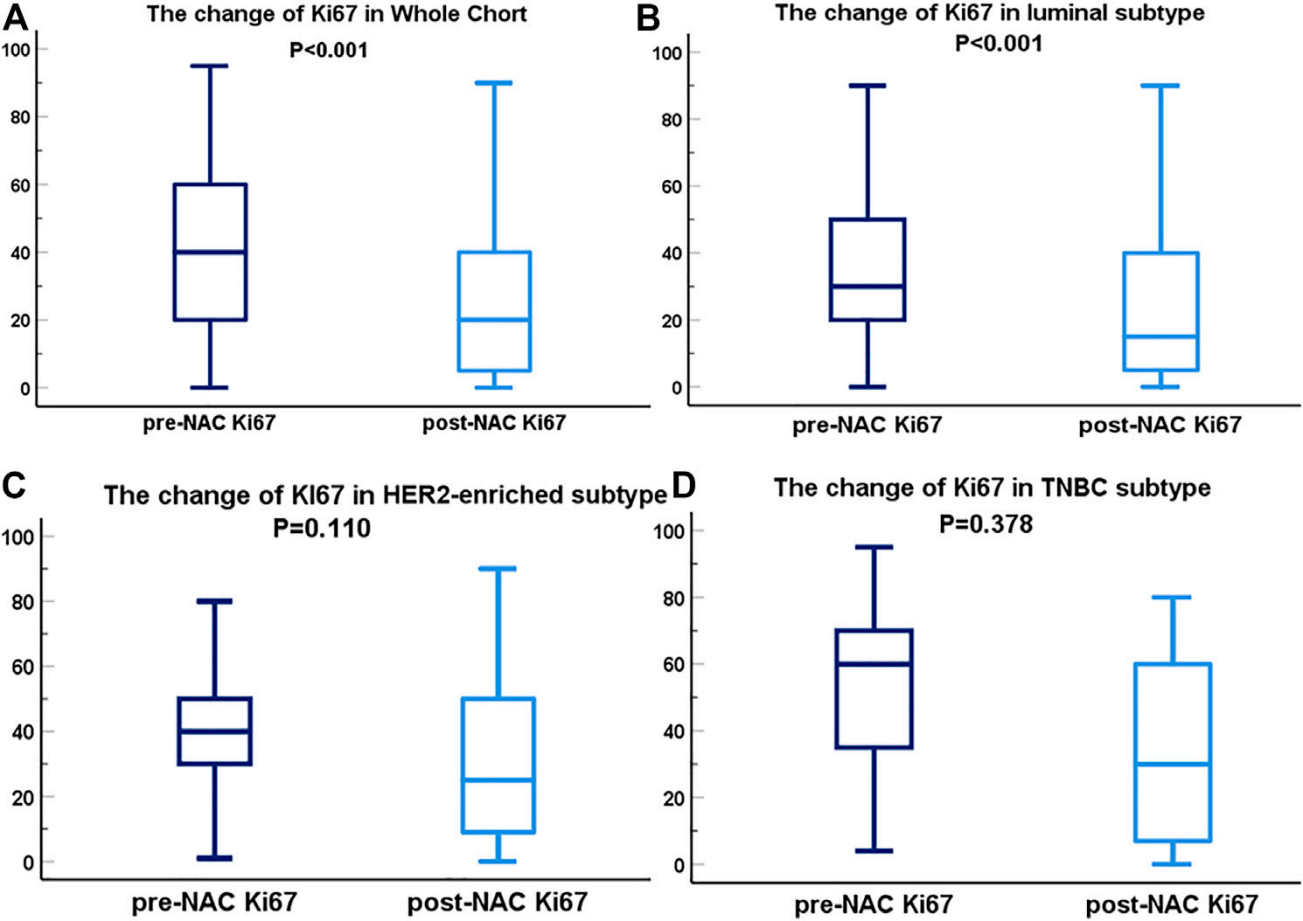
Box-plots of pre- and post-NAC Ki67 expression in diverse molecular subtypes; Ki67 variation in whole cohort **(A)**, Luminal subtype **(B)**, HER2 subtype **(C)** and TN breast cancer subtype **(D)**. The dark blue lines are on behalf of the box-plots of Ki67 before NAC. The light blue lines represent the box-plots of Ki67 after NAC. Abbreviations: pCR, pathological complete response; HER2, human epidermal growth factor receptor 2; TN breast cancer, triple negative breast cancer.

The average Ki67 status before NAC was 39.66% ± 22.61%. In comparison, the average value after NAC was 25.00% ± 22.91%. The downward trend of Ki67 before and after NAC is displayed in Figure 3 for the whole cohort (A), Luminal subtype (B), HER2-enriched subtype (C), and TN subtype (D). As shown in the figure, Ki67 change before and after NAC presented a statistical difference (*p* < 0.001) in Luminal subtype. However, in HER2-enriched and TN breast cancer, the difference of Ki67 represented no statistical significance. To evaluate the degree of Ki67 decline, we compared post-NAC and pre-NAC proliferation indices [define post-NAC Ki67 as A, define pre-NAC Ki67 as B, the computational formula was ΔKi67%=(A−B)÷B×100% , maintaining sign].

We next used ROC curve analysis to determine the optimal cut-off value for ΔKi67% . Performing the calculations on SPSS 26.0, we found that ΔKi67% had prognostic efficacy on survival outcomes among the full cohort. Based on the ROC calculation results in all patients, we defined an optimal cut-off of ΔKi67%≤−63% (*p* < 0.05). The ΔKi67% cut-off point in the Luminal B subtype was “−63%” (*p* = 0.047). Meanwhile the cut-off point in the TN breast cancer subtype was “−68%” (*p* = 0.009) (Table 4).

**TABLE 4:** The *p* values of AUCs in different subtype and corresponding *ΔKi67%* cut-off point.

**Molecular subtype^a^**	***p* Value of AUCs**	***ΔKi67%*** **cut-off point**
Luminal A	0.107	-
Luminal B	0.047	−63%
HER2-enriched	0.131	-
TNBC	0.009	−68%
Whole cohort	0.004	−63%

a Luminal A: (ER and PR positive, HER2 negative, “low” Ki-67, and a “low” recurrence risk based on multi-gene-expression assay results if available), Luminal B (“Luminal B-like (HER2 negative)”: ER positive, HER2 negative, and at least one of the following: “high” Ki-67, “negative or low” PR, or “high” recurrence risk based on multi-gene-expression assay if available. “Luminal B-like (HER2 positive)”: ER positive, HER2 over-expressed or amplified with any Ki-67, and any PR). HER2-enriched (HER2 over-expressed or amplified, HR absent) and TN (Negative HR and HER2).

AUC, area under curve.

Therefore, we defined a reduction of greater than 63% (ΔKi67%≤−63%) as ΔKi67% positive, and a reduction of less than 63% (ΔKi67>−63%) as ΔKi67% negative. Positive ΔKi67% status presented a larger magnitude of change for ΔKi67% between pre- and post- NAC, with negative status showing opposite. We used Chi-square tests to evaluate the relationship between demographic and pathological features and ΔKi67% status (Table 5). ΔKi67% -positive patients represented 37.1% of the full cohort. Histological type, number of chemotherapy cycles, type of chemotherapy regimen, clinical nodal status, molecular subtypes, pre- and post-NAC tumor size, and Ki67 all showed statistically significant relationship with ΔKi67% status (*p* values < 0.05).

**TABLE 5:** The univariate analysis of relation between basic characteristics with *ΔKi67%* status.

Parameter	ΔKi67% status		
	ΔKi67%≤−63% (Positive)	ΔKi67%>−63% (Negative)	*p* Value
Age at prognosis (years)			0.105
<40	24	59	
≥40	283	462	
BMI (kg/m^2^)			0.208
<18.9	26	42	
18.9–24.9	145	216	
>24.9	136	263	
Histological type			**<0.001**
IBC-NST	228	471	
Others	79	50	
Chemotherapy cycles			**<0.001**
≤2	48	151	
3–5	176	248	
>5	83	122	
Chemotherapy regimen			**<0.001**
Taxane-based	26	63	
Anthracycline-based	81	69	
Taxane + anthracycline	200	389	
Anti-HER2 therapy in patients with HER2-positive (*n* = 261)			0.265
Yes	22	27	
No	77	135	
Clinical tumor stage at diagnosis			**0.002**
T1	21	59	
T2	196	360	
T3/T4	90	102	
Post-NAC tumor size			0.169
<2 cm	174	260	
2–5 cm	120	235	
>5 cm	13	26	
Clinical nodal status			**0.011**
Positive	237	439	
Negative	70	82	
ER status^a^			0.884
Positive	196	330	
Negative	111	191	
PR positivity score^b^			0.532
<20%	197	323	
≥20%	110	198	
HER2			0.771
Positive	99	162	
Negative	161	286	
Unknown	47	73	
Pre-NAC Ki67			**<0.001**
<30%	67	186	
≥30%	240	335	
Post-NAC Ki67			**<0.001**
<30%	303	188	
≥30%	4	333	
Molecular subtypes^c^			**<0.001**
Luminal A	2	41	
Luminal B	195	294	
HER2-enriched	51	97	
TNBC	59	89	

a Positivity score<1% including negative status.

b Positivity score<20% including negative status.

c Luminal A: (ER and PR positive, HER2 negative, “low” Ki-67, and a “low” recurrence risk based on multi-gene-expression assay results if available), Luminal B (“Luminal B-like (HER2 negative)”: ER positive, HER2 negative, and at least one of the following: “high” Ki-67, “negative or low” PR, or “high” recurrence risk based on multi-gene-expression assay if available. “Luminal B-like (HER2 positive)”: ER positive, HER2 over-expressed or amplified with any Ki-67, and any PR). HER2-enriched (HER2 over-expressed or amplified, HR absent) and TN (Negative HR and HER2).

BMI, body mass index; NAC, neoadjuvant chemotherapy; IBC-NST, invasive breast carcinoma of no special Type; pCR, pathological complete response; ER, estrogen receptor; PR, progesterone receptor; HER2, human epidermal growth factor receptor 2; TNBC, triple negative breast cancer.

**FIGURE 4:**
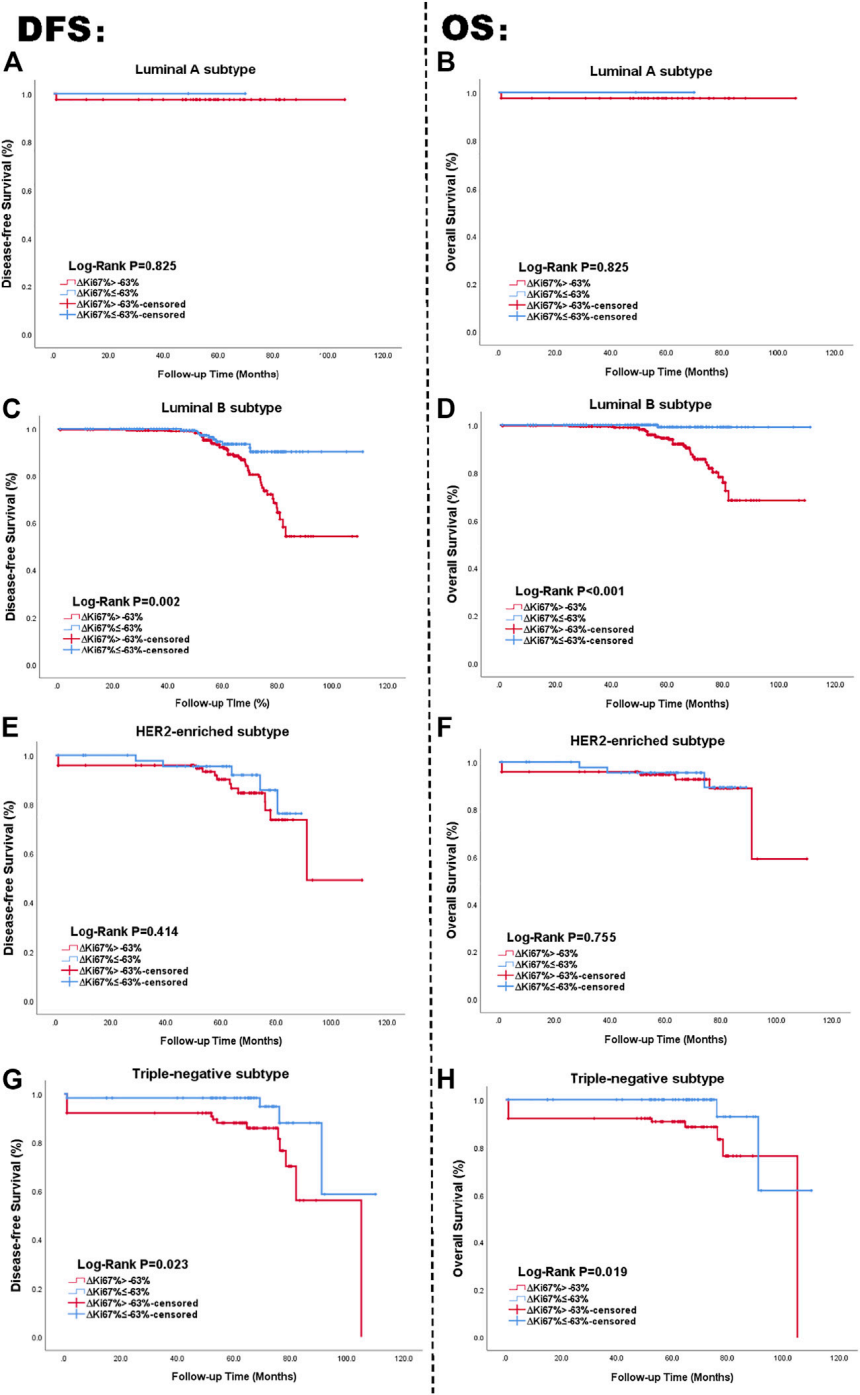
Kaplan Meier curve of DFS and OS in patients with ΔKi67% status; Luminal A subtype **(A, B)**, Luminal B subtype **(C, D)**, HER2-enriched subtype **(E, F)**, TN breast cancer subtype **(G, H)**. The blue lines are on behalf of achieving ΔKi67% -positive status after NAC. The red lines represented the ΔKi67% -negative status after NAC. The left side of the figure shows the relationship between ΔKi67% and DFS. The right side of the figure shows the relationship between ΔKi67% and OS. Abbreviations: HER2, human epidermal growth factor receptor 2; TN, triple negative breast cancer; DFS, disease-free survival; OS, overall survival.

In summary, ΔKi67% -positive status related with better survival outcomes. We used the Kaplan-Meier method to affirm the correlation between ΔKi67% status and survival outcomes in each molecular subtype. Survival curves of DFS and OS based on ΔKi67% status for the four subtypes are displayed in Figures 4A–H. Similar to Figure 2, the left side of the figure presents the Kaplan-Meier curves related with DFS and ΔKi67% , with the right side presenting the relationship between OS and ΔKi67% . As shown in the figure, the univariate log-rank test demonstrated ΔKi67% was statistically significantly related to DFS and OS in Luminal B and TN subtype, while it showed no definite effect in Luminal A and HER2-enriched subtypes (all *p* > 0.05).

**FIGURE 5:**
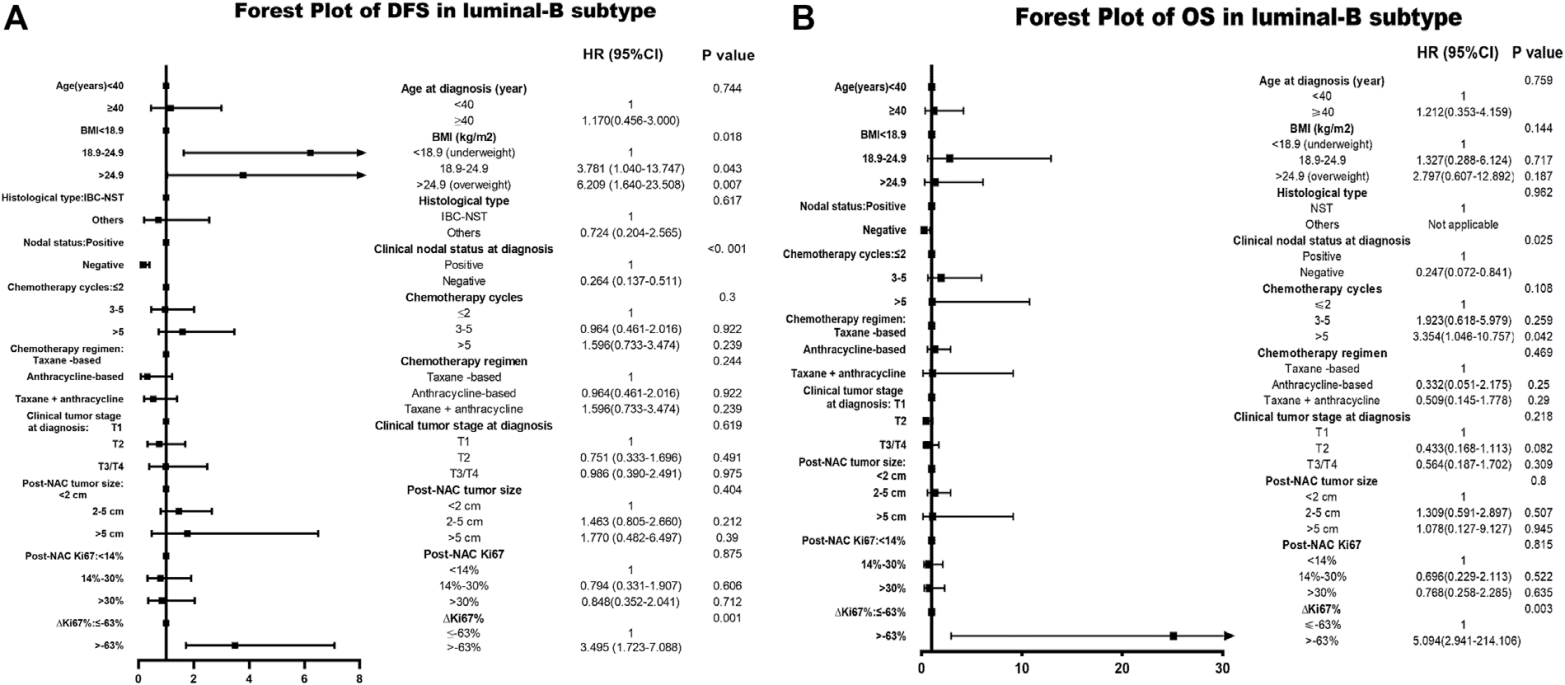
Forest plot of DFS and OS in Luminal B breast cancer; The left side of the figure is exhibiting the forest plot of DFS in Luminal B subtype. The right side of the figure presents the forest plot of OS in Luminal B subtype. The two forest plots both present the multivariate-analyses results; Abbreviations: BMI, body mass index; NAC, neoadjuvant chemotherapy; IBC-NST, invasive breast carcinoma of no special type; DFS, disease-free survival; OS, overall survival.

Based on the multivariate Cox analysis, ΔKi67% status is a significant independent prognostic predictor of survival outcome regardless of DFS and OS, with DFS-HR = 3.495 (95% CI 1.723–7.088, *p* = 0.001) and OS-HR = 23.024 (95% CI 2.956–179.333, *p* = 0.003) for the Luminal B subtype (Table 6, corresponding forest plot in Figure 5.

**TABLE 6:** The multivariate Cox analysis of ΔKi67% status in NAC-treated luminal-B subtype patients.

Parameter	Disease-free survival		Overall survival	
	HR (95% CI)	*p* Value	HR (95% CI)	*p* Value
Age at diagnosis (year)		0.741		0.718
<40	1.000		1.000	
≥40	1.171 (0.460–2.983)		1.251 (0.370–4.232)	
BMI (kg/m^2^)		**0.001**		0.172
<18.9 (underweight)	1.000		1.000	
18.9–24.9	4.425 (1.288–15.203)	**0.018**	1.347 (0.292–6.217)	0.702
>24.9 (overweight)	7.044 (2.015–24.625)	**0.002**	2.651 (0.591–11.899)	0.203
Histological type		0.625		0.961
IBC-NST	1.000		1.000	
Others	0.732 (0.209–2.565)		Not applicable	
Clinical nodal status at diagnosis		**<0.001**		**0.022**
Positive	1.000		1.000	
Negative	0.264 (0.137–0.511)		0.240 (0.071–0.817)	
Chemotherapy cycles		0.310		0.114
≤2	1.000		1.000	
3–5	0.975 (0.466–2.041)	0.947	1.916 (0.616–5.957)	0.261
>5	1.597 (0.736–3.464)	0.236	3.297 (1.033–10.529)	**0.044**
Chemotherapy regimen		0.216		0.474
Taxane-based	1.000		1.000	
Anthracycline-based	0.307 (0.081–1.154)	0.080	0.326 (0.051–2.076)	0.236
Taxane + anthracycline	0.538 (0.206–1.403)	0.205	0.540 (0.156–1.874)	0.332
Clinical tumor stage at diagnosis		0.585		0.211
T1	1.000		1.000	
T2	0.735 (0.327–1.651)	0.456	0.429 (0.167–1.102)	0.079
T3/T4	0.977 (0.389–2.456)	0.961	0.561 (0.185–1.697)	0.306
Post-NAC tumor size		0.383		0.796
<2 cm	1.000		1.000	
2–5 cm	1.473 (0.819–2.651)	0.196	1.306 (0.597–2.857)	0.504
>5 cm	1.760 (0.481–6.441)	0.393	1.056 (0.125–8.896)	0.960
Post-NAC Ki67		0.454		0.525
<30%	1.000		1.000	
≥30%	0.793 (0.432–1.456)		0.786 (0.375–1.649)	0.635
*ΔKi67%*		**0.001**		**0.003**
≤−63%	1.000		1.000	
>−63%	3.495 (1.723–7.088)		23.024 (2.956–179.333)	

BMI, body mass index; NAC, neoadjuvant chemotherapy; IBC-NST, invasive breast carcinoma of no special type.

Not only that, we tentatively continued to explore the subgroups of Luminal B patients based on the HER2-status. In Luminal B patients from our research, who with negative HER2 status were 256 (52.4%). The patients with positive HER2 status were 113 (23.3%). The patients with unknown HER2 status were 120 (24.5%). The Kaplan-Meier curves and multivariate-analysis results of relationship between *ΔKi67%* and survival outcome were attached in the Supplementary Presentation S1. Based on the statistical calculation, the analytical results of all subdivisions in Luminal B tumors fully supported the statistical significance of *ΔKi67%* (univariate and multivariate *p* < 0.05) except the DFS in HER2-positive Luminal B subtypes (univariate and multivariate *p* > 0.05). Combined, *ΔKi67%* was confirmed the statistically significant relationship with disease-free and overall survival outcome.

**FIGURE 6:**
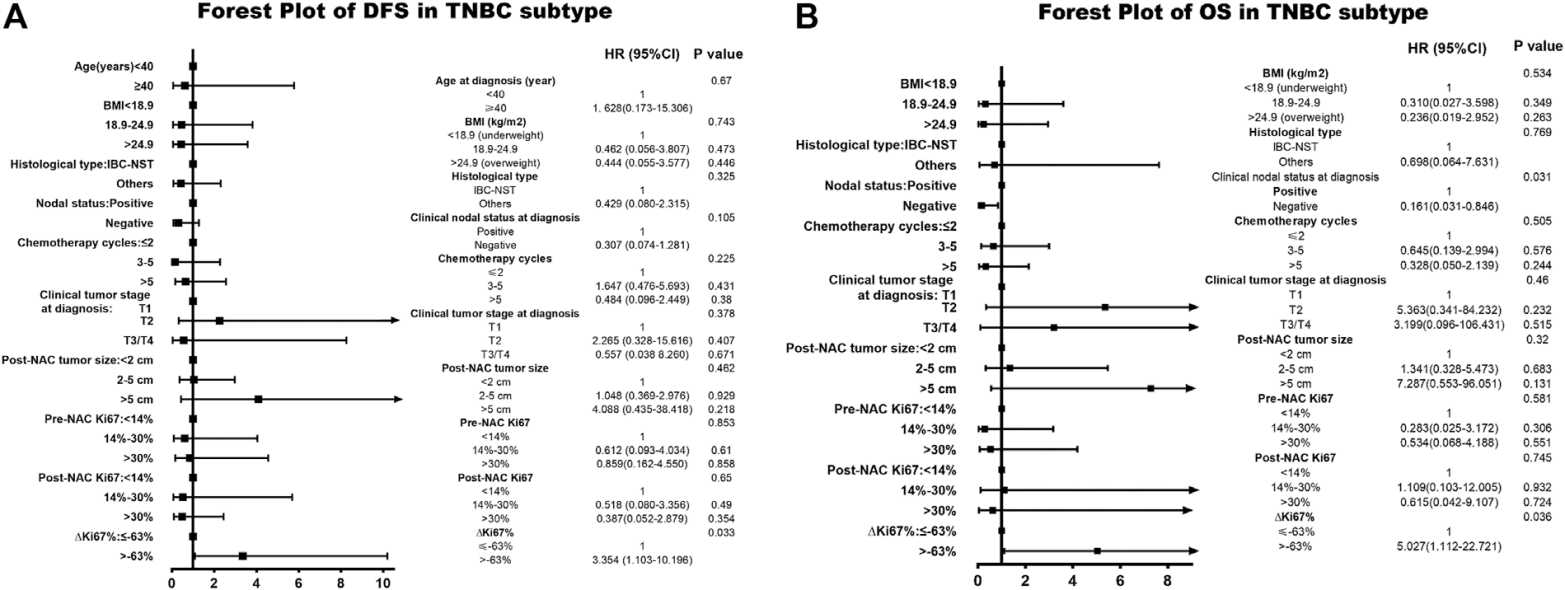
Forest plot of DFS and OS in TN breast cancer. The left side of the figure is exhibiting the forest plot of DFS in triple-negative subtype. The right side of the figure presents the forest plot of OS in triple-negative breast cancer. The two forest plots both present the multivariate-analyses results. Abbreviations: TN, triple negative; BMI, body mass index; NAC, neoadjuvant chemotherapy; IBC-NST, invasive breast carcinoma of no special type; DFS, disease-free survival; OS, overall survival.

Among patients with TN breast cancer, ΔKi67% status also provided meaningful survival forecasts on DFS (*p* = 0.023 < 0.05) and OS (*p* = 0.019 < 0.05) presented in Figure 4. The relationship between ΔKi67% status and survival outcomes in the TN breast cancer subtype was confirmed by multivariate Cox analysis as well, with DFS-HR = 3.354 (95% CI 1.103–10.196, *p* = 0.033) and OS-HR = 30.774 (95% CI 3.552–266.644, *p* = 0.002) (Table 7, corresponding forest plot in Figure 6). Two forest plots represented that negative ΔKi67% status is a valid indicator for better prognostics. We further used the Kaplan-Meier method to affirm the correlation between ΔKi67% status and survival outcomes in each molecular subtype. Survival curves based on ΔKi67% status for the four subtypes are displayed in Figure 6. ΔKi67% status shows statistically significant differences in Luminal B and TN breast cancer patients. The NRI value comparing the prognostic capacity between ΔKi67% status and pCR in TN breast cancer subtype was 0.685 (95% CI 0.3336–1.0294, *p* < 0.001), also supporting our conclusions. In both Figures 5, 6 ΔKi67% had a statistically significant relationship with prognostic outcome.

**TABLE 7:** The multivariate Cox analysis of ΔKi67% status in NAC-treated TNBC subtype patients.

Parameter	Disease-free survival		Overall survival	
	HR (95% CI)	*p* Value	HR (95% CI)	*p* Value
Age at diagnosis (year)		0.715		0.988
<40	1.000		1.000	
≥40	1.628 (0.173–15.306)		Not applicable	
BMI (kg/m^2^)		0.743		0.154
<18.9 (underweight)	1.000		1.000	
18.9–24.9	0.462 (0.056–3.807)	0.473	0.076 (0.005–1.056)	0.055
>24.9 (overweight)	0.444 (0.055–3.577)	0.446	0.179 (0.018–1.834)	0.179
Histological type		0.325		0.905
IBC-NST	1.000		1.000	
Others	0.429 (0.080–2.315)		1.154 (0.109–12.233)	
Clinical nodal status at diagnosis		0.105		**0.027**
Positive	1.000		1.000	
Negative	0.307 (0.074–1.281)		0.132 (0.022–0.798)	
Chemotherapy cycles		0.225		0.469
≤2	1.000		1.000	
3–5	1.647 (0.476–5.693)	0.431	0.879 (0.203–3.811)	0.863
>5	0.484 (0.096–2.449)	0.380	0.315 (0.043–2.294)	0.254
Clinical tumor stage at diagnosis		0.378		0.308
T1	1.000		1.000	
T2	2.265 (0.328–15.616)	0.407	9.973 (0.513–194.007)	0.129
T3/T4	0.557 (0.038 8.260)	0.671	6.405 (0.167–246.220)	0.319
Post-NAC tumor size		0.462		0.405
<2 cm	1.000		1.000	
2–5 cm	1.048 (0.369–2.976)	0.929	0.939 (0.217–4.060)	0.933
>5 cm	4.088 (0.435–38.418)	0.218	5.091 (0.414–62.551)	0.204
Pre-NAC Ki67		0.137		0.089
<30%	1.000		1.000	
≥30%	4.442 (0.624–31.635)		8.686 (0.722–104.507)	
Post-NAC Ki67		0.027		**0.032**
<30%	1.000		1.000	
≥30%	6.880 (1.238–38.224)		7.221 (1.181–44.133)	
∆Ki67%		0.033		**0.002**
≤−63%	1.000		1.000	
>−63%	3.354 (1.103–10.196)		30.774 (3.552–266.644)	

BMI, body mass index; NAC, neoadjuvant chemotherapy; IBC-NST, invasive breast carcinoma of no special type.

## Discussion

NAC is currently widely applied to shrink tumors and decrease carcinoma volume, allowing patients to preserve breasts or become operable [15]. Moreover, the pathological response to NAC is also beneficial for optimizing chemotherapy regimens and predicting relapse possibility and survival outcomes. Patients with pCR to NAC show improved rates of relapse and better survival [33,34].

Many studies have verified correlations between the degree of Ki67 reduction and pathological response to NAC [10,35,36], yet controversies exist regarding the relationships between Ki67 index, pathological response, and survival grates. Some studies only find significant Ki67 proliferation index differences when comparing pre-NAC with post-surgery in Luminal subtypes [37,38] while other studies have demonstrated that Ki67 reduction also plays a role in TN breast cancer [39,40]. While a separate investigation mentioned ΔKi67% , it only discussed its prognostic role and predictive value within 90 Luminal subtype patients with neoadjuvant letrozole-based treatment without classifying it more broadly [20].

In this retrospective real-world study, we analyzed basic demographic and pathological characteristics relative to pCR following to NAC. We confirmed that breast cancer pathological subtype, chemotherapy cycle number, maximum tumor diameter after NAC, nodal state at diagnosis, and Ki67 index pre-NAC and post-NAC all presented statistically significant differences. Furthermore, ER and PR status and molecular subtypes all showed significant effects on pCR rate, as verified in previous studies [41].

As mentioned above, pCR rate ranged from 15 to 20% in previous studies [7–9]. Patients who achieved pCR following NAC represented 16.7% of our cohort, a relatively small portion of the total patients. This implies that pCR status increases the specificity of survival outcome predictions but lowered the sensitivity. Many patients were eliminated in the evaluation system, especially those with the Luminal B subtype who represent the largest portion of all breast cancer patients. This is consistent with previous large trials showing pCR rates to have limited prognostic value in patients with Luminal B subtype [11,12]. ∆Ki67% status could help improve this deficiency.

Our study has many strengths. Our fundamental statistical data of post-NAC Ki67 is in accordance with previous research about the relevance of clinical response to NAC and prognostic value [12,24,25]. Subtracting pre- and post-NAC Ki67 is insufficient to account for all situations, therefore we used ΔKi67% as a rational solution. ΔKi67% is an indicator capable of considering the extent of Ki67 changes in all individuals. Since achievement of pCR is not a useful prognostic indicator in the Luminal B subtype, the field currently lacks efficient parameters to predict outcome and assist in clinical decisions makings for these patients [11,12].

∆Ki67% is a useful indicator for more than just Luminal B subtype patients. In patients with TN breast cancer, pCR rate and ΔKi67% status both predicted survival outcome with statistical significance. The multivariate analysis confirmed that ΔKi67% status independently predicted long-term outcomes as well. ΔKi67% status may be capable to aid with NAC regimen modification with pCR status in the TN subtype. In Luminal B subtypes, we made the research based on different HER2-status subgroups. Nearly all results of subgroups support our conclusions regardless of DFS and OS. Except the HER2-positive Luminal B tumors, the DFS *p* value of univariate and multivariate analysis is over 0.05. Meanwhile, considering the function of ΔKi67% in HER2-enriched subtype, it also prompted that HER2(human epidermal growth factor receptor 2) could influence the predictive efficacy of Ki67. The results enlightened us to collect relative data and dig the thoughts deeper.

This study has inherent limitations. Missing data is a common issue in most retrospective single center studies. Hence, we excluded patients whose information was incomplete or inadequate to be incorporated in the study cohort. The second limitation when using Ki67 staining and assessment is lack of stable measurement results [13,14]. To account for this, we adopted the ‘hottest-spot’ method and performed pathological assessments strictly following international guidelines to improve reproducibility. In the future, artificial intelligence in precision pathology could dramatically improve this method.

In this study, we demonstrate that ΔKi67% status serves as an independent prognostic factor in Luminal B subtype patients. According to the POETIC clinical phase-3 trial, Ki67 variation in women with operable ER-positive primary breast cancer after preoperative and perioperative aromatase inhibitor (POAI) therapy assisted in deciding further adjuvant endocrine therapy and chemotherapy [19]. This indicates that ΔKi67% could fill the current gap for predicting prognostic outcomes in Luminal B subtype patients and assist in further clinical treatment decisions to help modify further adjuvant regimens.

## Conclusion

In this study, we validated that the extent of Ki67 change before and after NAC, termed ΔKi67% , associates with patient survival outcomes across subtypes. Our statistical calculations defined a cut-off value for ΔKi67% of (−63%). We confirmed that ΔKi67% status presents an independent prognostic prediction indicator for long-term outcome in Luminal B and TN breast cancer subtypes. As pCR achievement is not a statistically significant predictor for Luminal B subtype patients, ΔKi67% status may fill this clinical vacancy, assisting with measuring efficacy of neoadjuvant therapy and providing data for adjuvant therapy adjustment [42].

## Data Availability

The raw data supporting the conclusions of this article will be made available by the authors, without undue reservation.

## Supplementary Material

Supplementary Figure S1 The Kaplan-Meier curves of ΔKi67% in whole cohort. The Supplementary Figure S1 exhibits the results of univariate analysis, Kaplan-Meier method. The left side of the picture presents the relationship between the ΔKi67% status and DFS. The right side of the picture shows the relationship between the ΔKi67% status and OS. The red lines represent the ΔKi67% -positive status after NAC. The blue lines represent the ΔKi67% -negative status after NAC. Abbreviations: DFS, disease-free survival; OS, overall survival.

Supplementary Figure S2 Representative immunohistochemical staining of Ki67. Ki67<30% **(A, B)**, Ki67≥30% **(C, D)**.

Supplementary Presentation S1 The results of univariate and multivariate analyses in subdivisions of Luminal B patients. The PDF file contains the Kaplan-Meier curves and tabular-forms of multivariate results regardless of DFS and OS. Luminal B subtype was sorted into four subdivisions: negative HER2 status, positive HER2 status, luminal B subtype excluded HER2-positive status (containing negative and unknown HER2 status) and luminal B subtype excluded HER2-negative status (containing positive and unknown HER2 status). The blue lines are on behalf of achieving ΔKi67% -positive status after NAC. The red lines represented the ΔKi67% -negative status after NAC. The left side of the figure shows the relationship between ΔKi67% and DFS. The right side of the figure shows the relationship between ΔKi67% and OS; Abbreviations: BMI, body mass index; NAC, neoadjuvant chemotherapy; IBC-NST, invasive breast carcinoma of no special type; pCR, pathological complete response; ER, estrogen receptor; PR, progesterone receptor; HER2, human epidermal growth factor receptor 2; TNBC, triple negative breast cancer; DFS, disease-free survival; OS, overall survival.

## Abbreviations

AUC, area under the curve; BMI, body mass index; CI, confidence intervals; DFS, disease-free survival; ER, estrogen receptor; FISH, fluorescent *in situ* hybridization; HR, Hazard Ratio; HR, hormone receptors; HER2, human epidermal growth receptor-2; IBC-NST, invasive breast carcinoma of no special type; IRB, institutional review board; IHC, Immunohistochemical; NAC, neoadjuvant chemotherapy; NRI, net reclassification improvement; OS, overall survival; PR, progesterone receptor; pCR, pathological complete response; ROC, operating characteristic curve; TN, triple negative.
