# Supplementary figures and images for: Quantification of Ki67 Change as a Valid Prognostic Indicator of Luminal B Type Breast Cancer After Neoadjuvant Therapy
Source: Pathol Oncol Res. 2021 Dec 20;27:1609972. doi: 10.3389/pore.2021.1609972 (PMC8722379; doi:10.3389/pore.2021.1609972)

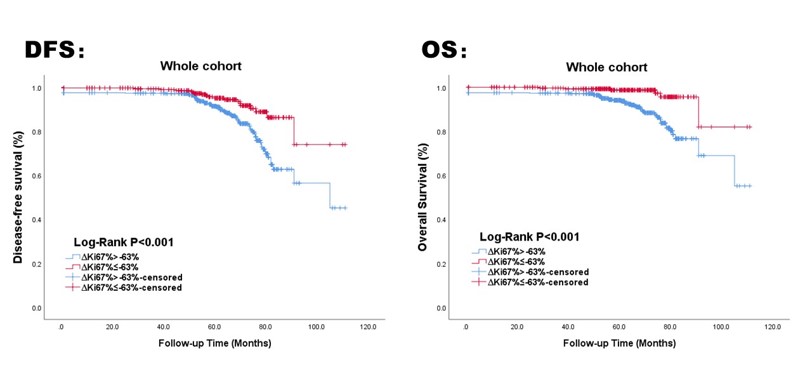

Supplement: Supplementary file 1 [file Image1.TIFF]

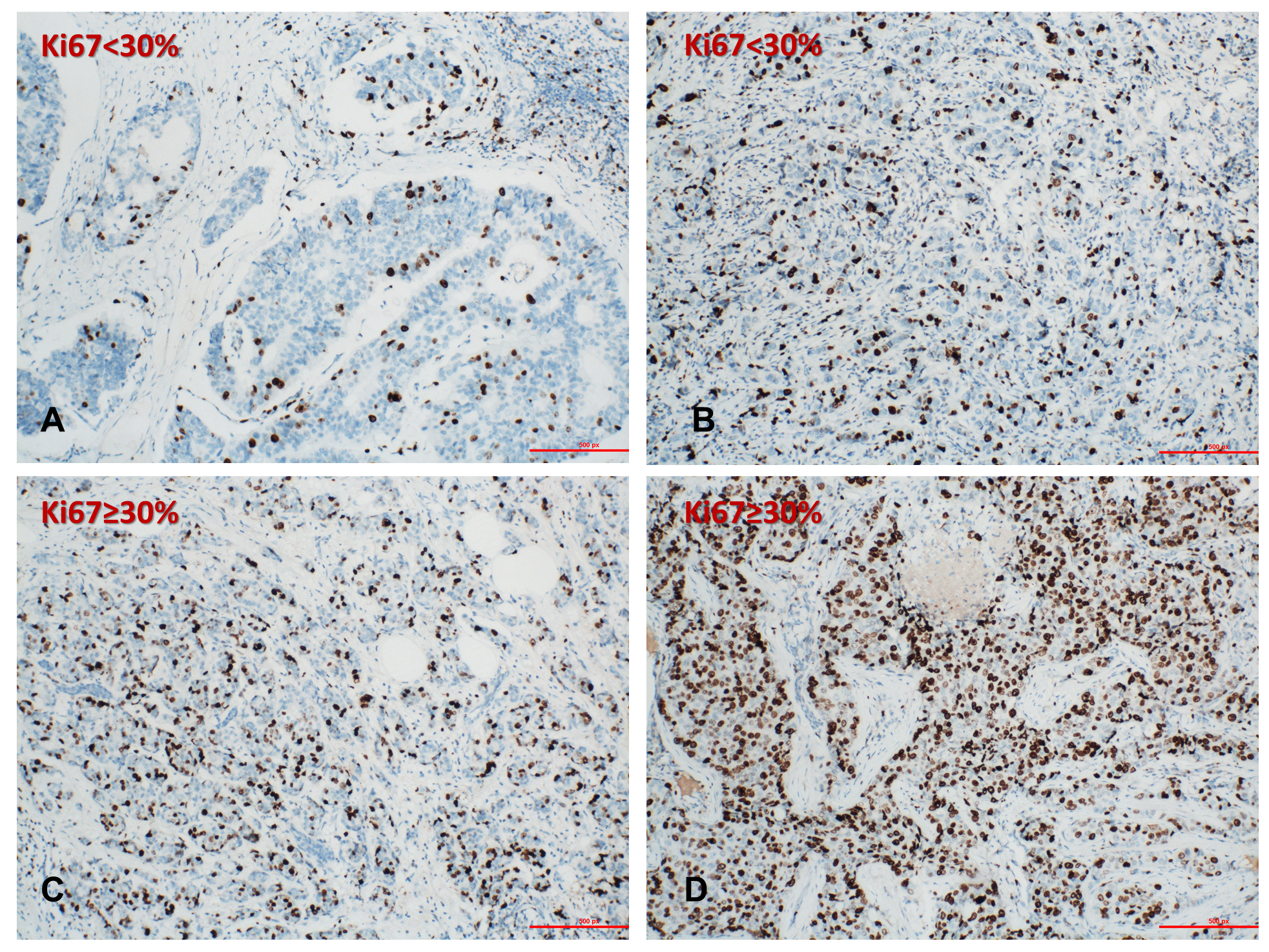

Supplement: Supplementary file 3 [file Image2.TIFF]
