# Supplementary material for: Quantification of Ki67 Change as a Valid Prognostic Indicator of Luminal B Type Breast Cancer After Neoadjuvant Therapy
Source: Pathol Oncol Res. 2021 Dec 20;27:1609972. doi: 10.3389/pore.2021.1609972 (PMC8722379; doi:10.3389/pore.2021.1609972)
Supplement: Supplementary file 2 [file Table1.docx]

**Additional Table 1: the multivariate Cox analysis of** $\boldsymbol{\Delta}\boldsymbol{Ki}\boldsymbol{67\%}$ **status in whole patients**

| **Parameter** | **Disease-free survival** | | **Overall survival** | |
| --- | --- | --- | --- | --- |
|  | **HR (95% CI)** | **P value** | **HR (95% CI)** | **P value** |
| **Age at diagnosis (year)** |  | 0.251 |  | 0.152 |
| <40 | 1.000 |  | 1.000 |  |
| ≥40 | 1.585 (0.722-3.476) |  | 2.378 (0.726-7.789) |  |
| **BMI (kg/m2)** |  | **0.012** |  | **0.026** |
| <18.9 (underweight) | 1.000 |  | 1.000 |  |
| 18.9-24.9 | 2.181(0.951-5.003) | **0.066** | 1.097 (0.414-2.903) | 0.853 |
| >24.9 (overweight) | 3.068 (1.326-7.099) | **0.009** | 2.267(0.880-5.841) | 0.090 |
| **Histological type** |  | 0.898 |  | 0.715 |
| IBC-NST | 1.000 |  | 1.000 |  |
| Others | 1.052 (0.486-2.277) |  | 0.817 (0.276-2.417) |  |
| **Clinical nodal status at diagnosis** |  | **0.005** |  | 0.056 |
| Positive | 1.000 |  | 1.000 |  |
| Negative | 0.467(0.275-0.793) |  | 0.503 (0.249-1.016) |  |
| **Chemotherapy cycles** |  | 0.937 |  | 0.542 |
| ≤2 | 1.000 |  | 1.000 |  |
| 3-5 | 1.096 (0.655-1.835) | 0.727 | 1.267 (0.650-2.471) | 0.488 |
| >5 | 1.037 (0.576-1.867) | 0.904 | 1.527 (0.721-3.235) | 0.269 |
| **Chemotherapy regimen** |  | 0.238 |  | 0.708 |
| Taxane -based | 1.000 |  | 1.000 |  |
| Anthracycline-based | 0.442 (0.171-1.143) | 0.092 | 0.997 (0.273-3.640) | 0.996 |
| Taxane + anthracycline | 0.744 (0.390-1.420) | 0.369 | 1.346 (0.499-3.631) | 0.577 |
| **Clinical tumor stage at diagnosis** |  | 0.714 |  | 0.421 |
| T1 | 1.000 |  | 1.000 |  |
| T2 | 1.083 (0.543-2.160) | 0.821 | 0.756 (0.337-1.695) | 0.497 |
| T3/T4 | 1.323 (0.595-2.945) | 0.492 | 1.152 (0.451-2.944) | 0.768 |
| **Post-NAC tumor size** |  | 0.993 |  | 0.928 |
| <2 cm | 1.000 |  | 1.000 |  |
| 2-5 cm | 0.974 (0.625-1.517) | 0.906 | 0.957 (0.539-1.700) | 0.881 |
| >5 cm | 0.985 (0.336- 2.887) | 0.979 | 0.746 (0.165-3.373) | 0.703 |
| **ER status ^a^** |  | **0.048** |  | **0.029** |
| Positive | 1.000 |  | 1.000 |  |
| Negative | 3.448 (1.009-11.777) |  | 4.797 (1.173-19.607) |  |
| **PR positivity score ^b^** |  | 0.944 |  | 0.220 |
| <20% | 1.000 |  | 1.000 |  |
| ≥20% | 1.020 (0.587-1.772) |  | 1.596 (0.757-3.365) |  |
| **HER2** |  | 0.375 |  | 0.918 |
| Negative | 1.000 |  | 1.000 |  |
| Positive | 1.628 (0.835-3.174) | 0.152 | 1.195 (0.490-2.916) | 0.695 |
| Unknown | 1.251 (0.662-2.366) | 0.491 | 1.117 (0.494-2.527) | 0.791 |
| **Pre-NAC Ki67** |  | 0.660 |  | 0.383 |
| <30% | 1.000 |  | 1.000 |  |
| ≥30% | 1.126 (0.662-1.915) |  | 1.372 (0.675-2.787) |  |
| **Post-NAC Ki67** |  | 0.853 |  | 0.682 |
| <30% | 1.000 |  | 1.000 |  |
| ≥30% | 0.950 (0.551-1.638) |  | 0.866 (0.436-1.720) | 0.823 |
| **Molecular subtypes ^c^** |  | **0.013** |  | 0.075 |
| Luminal A | 1.000 |  | 1.000 |  |
| Luminal B | 6.477(0.892-47.027) | 0.065 | 4.448 (0.600-32.944) | 0.144 |
| HER2-enriched | 1.567(0.156-15.734) | 0.703 | 0.909 (0.075-10.969) | 0.940 |
| TNBC | 1.655(0.164-16.661) | 0.669 | 1.252(0.105-14.903) | 0.859 |
| **∆Ki67%** |  | **<0. 001** |  | **<0. 001** |
| ≤-63% | 1.000 |  | 1.000 |  |
| >-63% | 2.829 (1.692-4.730) |  | 5.775 (2.459-13.561) |  |

BMI body mass index, NAC neoadjuvant chemotherapy, IBC-NST invasive breast carcinoma of no special type, pCR pathological complete response, ER estrogen receptor, PR progesterone receptor, HER2 human epidermal growth factor receptor 2, TNBC: triple negative breast cancer

a Positivity score<1% including negative status

b Positivity score<20% including negative status

c Luminal A: (ER and PR positive, HER2 negative, “low” Ki-67, and a “low” recurrence risk based on multi-gene-expression assay results if available), Luminal B (“Luminal B-like (HER2 negative)”: ER positive, HER2 negative, and at least one of the following: “high” Ki-67, “negative or low” PR, or “high” recurrence risk based on multi-gene-expression assay if available. “Luminal B-like (HER2 positive)”: ER positive, HER2 over-expressed or amplified with any Ki-67, and any PR). HER2-enriched (HER2 over-expressed or amplified, HR absent) and TN (Negative HR and HER2).
